# Supplementary material for: Napthoquinones from Neocosmospora sp.—Antibiotic Activity against Acidovorax citrulli, the Causative Agent of Bacterial Fruit Blotch in Watermelon and Melon
Source: J Fungi (Basel). 2021 May 8;7(5):370. doi: 10.3390/jof7050370 (PMC8151544; doi:10.3390/jof7050370)
Supplement: Supplementary file 1 [file jof-07-00370-s001.zip › jof-1182529-supplementary.pdf]

Supplementary Materials

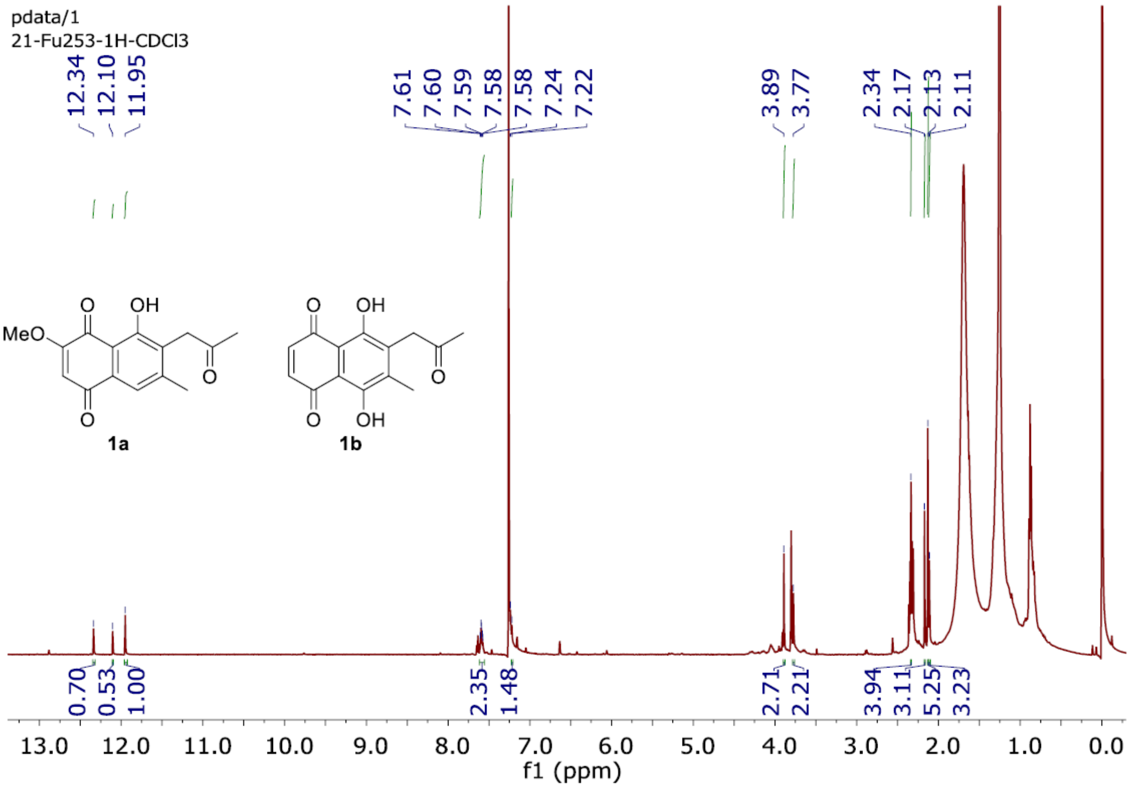

Figure S1. 1H NMR spectrum of mixture of compound **1a** and **1b** in CDCl3.

**Sample Name**	21_FU253_NEG	**Position**	P1-F2	**Instrument Name**	Instrument 1
**User Name**		**Inj Vol**	1	**InjPosition**	
**Sample Type**	Sample	**IRM Calibration Status**	Success	**Data Filename**	21_FU253_NEG.d
**ACQ Method**	NEG_PURE_50MEOHG_50FORMIC ACID_01P.m	**Comment**		**Acquired Time**	11/3/2020 5:35:06 PM (UTC+07:00)

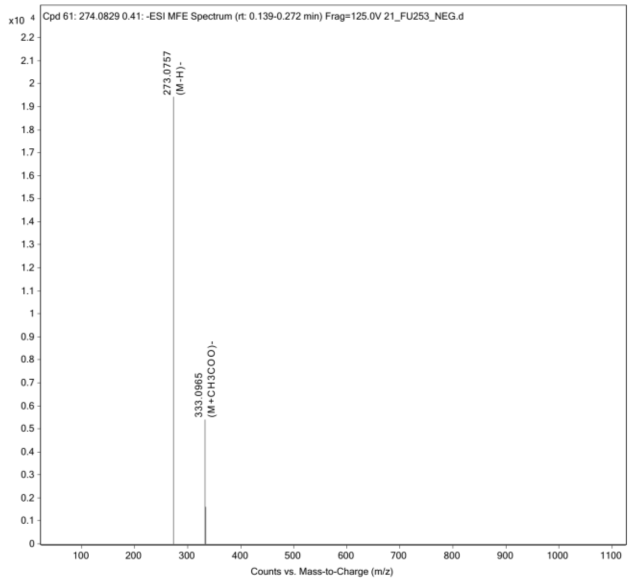

Figure S2. Negative mode ESI-MS of compound **1a**.

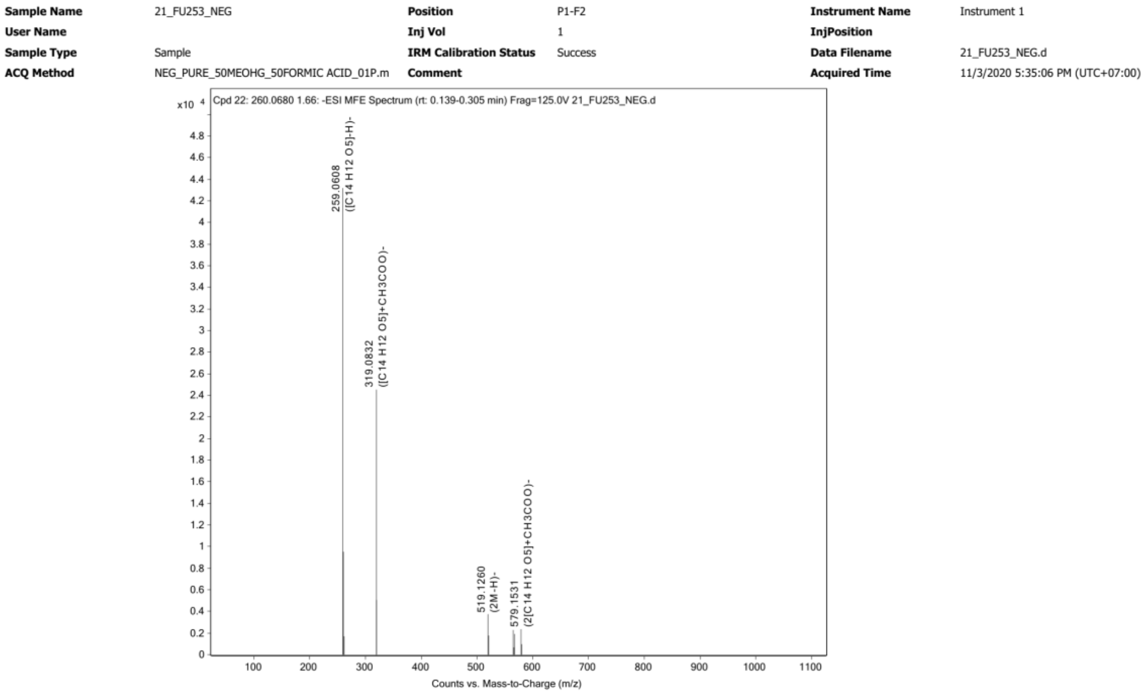

Figure S3. Negative mode ESI-MS of compound **1b**.

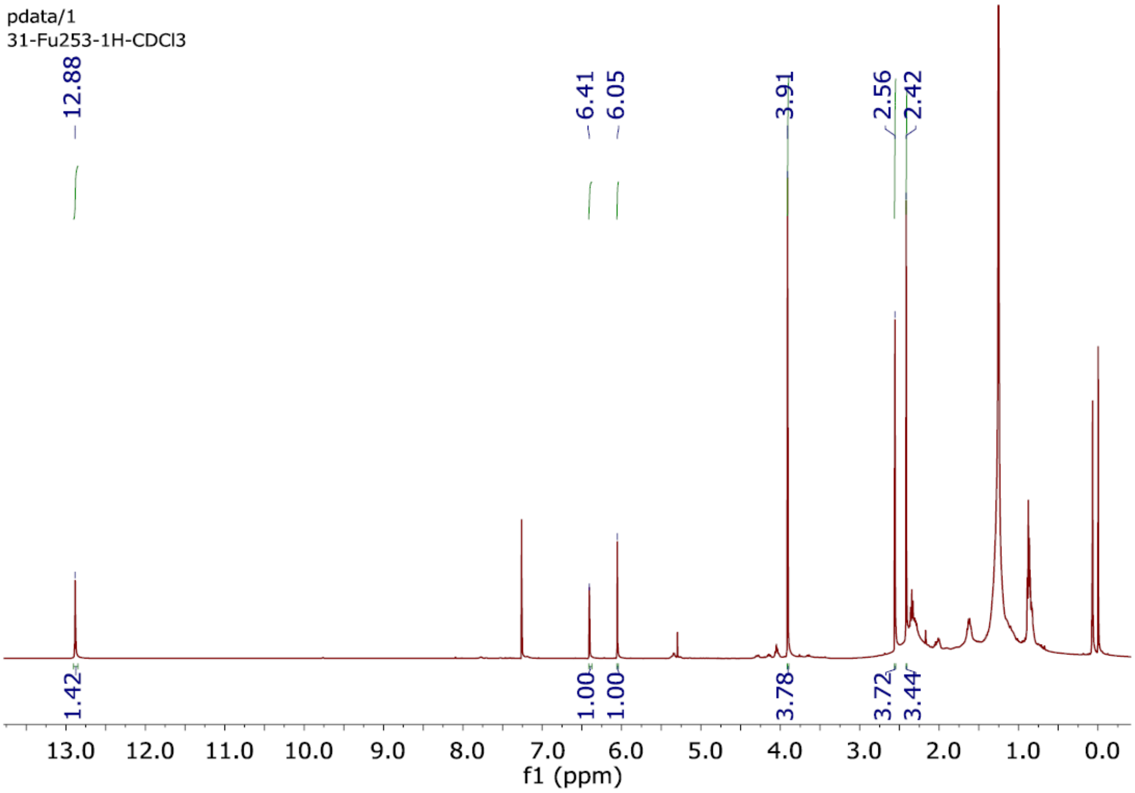

Figure S4. 1H NMR spectrum of mixture of compound 2 in CDCl3.

**Sample Name**	31_FU253_POS	**Position**	P1-F4	**Instrument Name**	Instrument 1
**User Name**		**Inj Vol**	1	**InjPosition**	
**Sample Type**	Sample	**IRM Calibration Status**	Success	**Data Filename**	31_FU253_POS.d
**ACQ Method**	POS_PURE_50MEOHG_50FORMIC ACID_01P.m	**Comment**		**Acquired Time**	11/3/2020 5:23:29 PM (UTC+07:00)

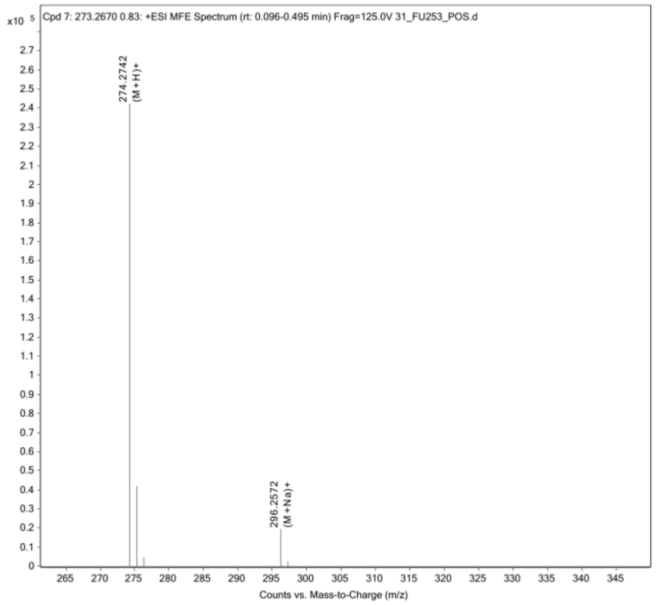

Figure S5. Positive mode ESI-MS of compound 2.

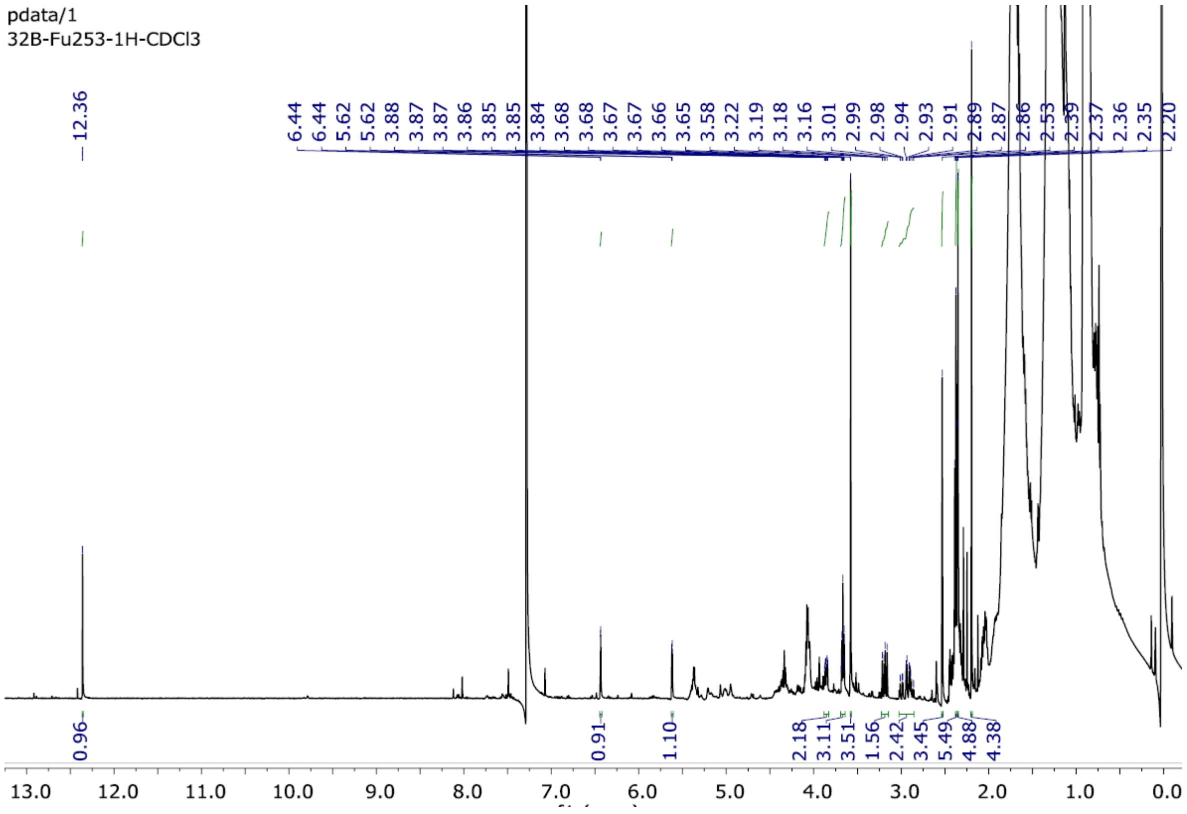

Figure S6. 1H NMR spectrum of compound 3 in CDCl3.

**Sample Name**	32B_FU_POS	**Position**	P1-F3	**Instrument Name**	In
**User Name**		**Inj Vol**	1	**InjPosition**	
**Sample Type**	Sample	**IRM Calibration Status**	Success	**Data Filename**	32
**ACQ Method**	POS_PURE_50MEOHG_50FORMIC ACID_01P.m	**Comment**		**Acquired Time**	11

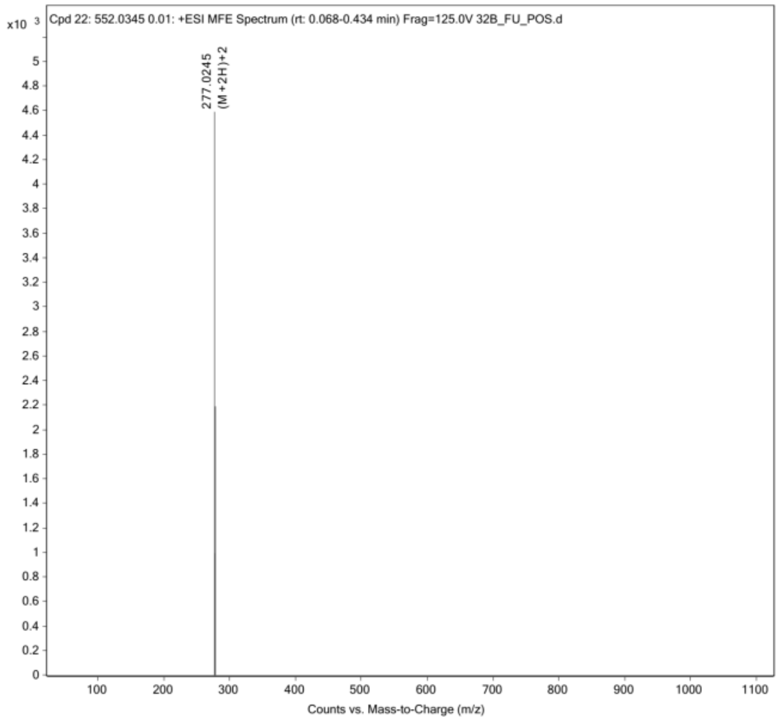

Figure S7. Positive mode ESI-MS of compound 3.
